# Renal Hemangioblastoma with Mixed Mullerian tumour of endometrium: A tale of two rare primary tumours

**DOI:** 10.1186/s12957-020-01929-1

**Published:** 2020-07-06

**Authors:** Aparna Setia, Devender Kumar, Lovenish Bains, Pallavi Sharma, Anjali Tempe, Varuna Mallya

**Affiliations:** 1 Department of Obstetrics and Gynaecology, Maulana Azad Medical College, New Delhi, India; 2 Department of Surgery, Maulana Azad Medical College, New Delhi, India; 3 Department of Pathology, Maulana Azad Medical College, New Delhi, India

**Keywords:** Endometrial cancer, Mixed Mullerian tumour (MMT), Renal hemangioblastoma (RH), Renal cell carcinoma (RCC)

## Abstract

**Introduction:**

Renal hemangioblastoma (RH) is a very rare benign tumour. Hemangioblastoma most commonly occurs in the central nervous system (CNS), and only few cases of RH have been reported as they occur most commonly as asymptomatic masses found incidentally. Mixed Mullerian tumour (MMT) of the uterus is a rarer and aggressive form of uterine malignancy. The detection of two primary rare tumours incidentally is a rare entity.

**Case presentation:**

A 50-year-old female presented with abnormal uterine bleeding which on endometrial sampling was diagnosed as a rare variety of endometrial cancer, i.e. MMT or uterine carcinosarcoma. On preoperative imaging, a renal mass was also detected which was highly vascular and was mimicking renal cell carcinoma (RCC). Fine needle aspiration cytology (FNAC) was done from the renal mass to differentiate between RCC and metastasis, but it showed only blood cells. Patient underwent staging laparotomy for endometrial cancer and frozen section examination of the renal mass which was inconclusive with few atypical cells, and thus, patient underwent radical nephrectomy too. Histopathological examination revealed it to be a RH which is a very rare benign tumour.

**Discussion:**

RH is a rare benign tumour which does not require any treatment in majority of the patients. Only 26 cases of RH outside the CNS have been reported till date. MMT is a rare aggressive uterine tumour with an incidence of 1–2 % of all uterine neoplasms, which metastasizes early, and thus, early identification and treatment is the key. RH needs to be differentiated from RCC to avoid over treatment. Morphological findings are similar in RCC and RH; preoperative FNAC, PET scan, and intraoperative frozen section can be utilized to differentiate the two, in well-circumcised tumours and high index of suspicion. Occurrence of renal mass as an incidental finding in the preoperative work up of uterine malignancy directed us to the differentials of metastasis or another histologically distinct primary tumour. The presence of two rare primary tumours, i.e. RH and MMT in the same patient which are unrelated, is a rare entity.

## Introduction

Hemangioblastoma, a rare benign tumour, is more commonly associated with Von Hippel-Lindau (VHL) syndrome, and its most common site is CNS. Hemangioblastoma can occur in other sites such as the kidney. To date, only 26 cases of a renal hemangioblastoma (RH) have been reported in literature; thus, it is a very rare benign tumour [1, 2] occurring as a sporadic tumour and is mostly as an incidental finding. MMT of uterus, also known as carcinosarcoma, is also a rare and aggressive variety of uterine cancer with high chances of early metastasis. The incidence of MMT is 1 to 4 per 100,000 women [3, 4]. We present a rare case of two rare primary tumours occurring in the same patient.

## Case presentation

A 50-year-old woman presented to our Gynecology OPD with chief complaints of bleeding per vaginum for 4 months, pain lower abdomen, and dyspareunia for 2 months. Patient had regular menstrual cycles lasting for 6 days, occurring every 28 days till 4 months back when she developed vaginal bleeding, associated with passage of clots, pain, and sexual discomfort. She never used oral contraceptive pills or any other method of contraception. She has two children, both normal vaginal delivery with last childbirth 17 years back. Patient was detected with hypothyroidism 2 years ago and was maintaining euthyroid status on thyroxine 50 μg daily. There was no significant history of any genital, colonic, or renal malignancy in her family.

On examination, patient was average built, afebrile, pulse 76/min, blood pressure 110/74 mmHg, and mild pallor was present. Abdomen was soft and did not reveal any mass. Per speculum examination revealed hypertrophied cervix and slight bleeding through external os. On per vaginal examination, cervix appeared firm and uterus was bulky, firm, and mobile with no palpable adnexal mass through fornices. Per rectal examination, she had normal rectal mucosa and soft parametrium.

**Fig. 1:**
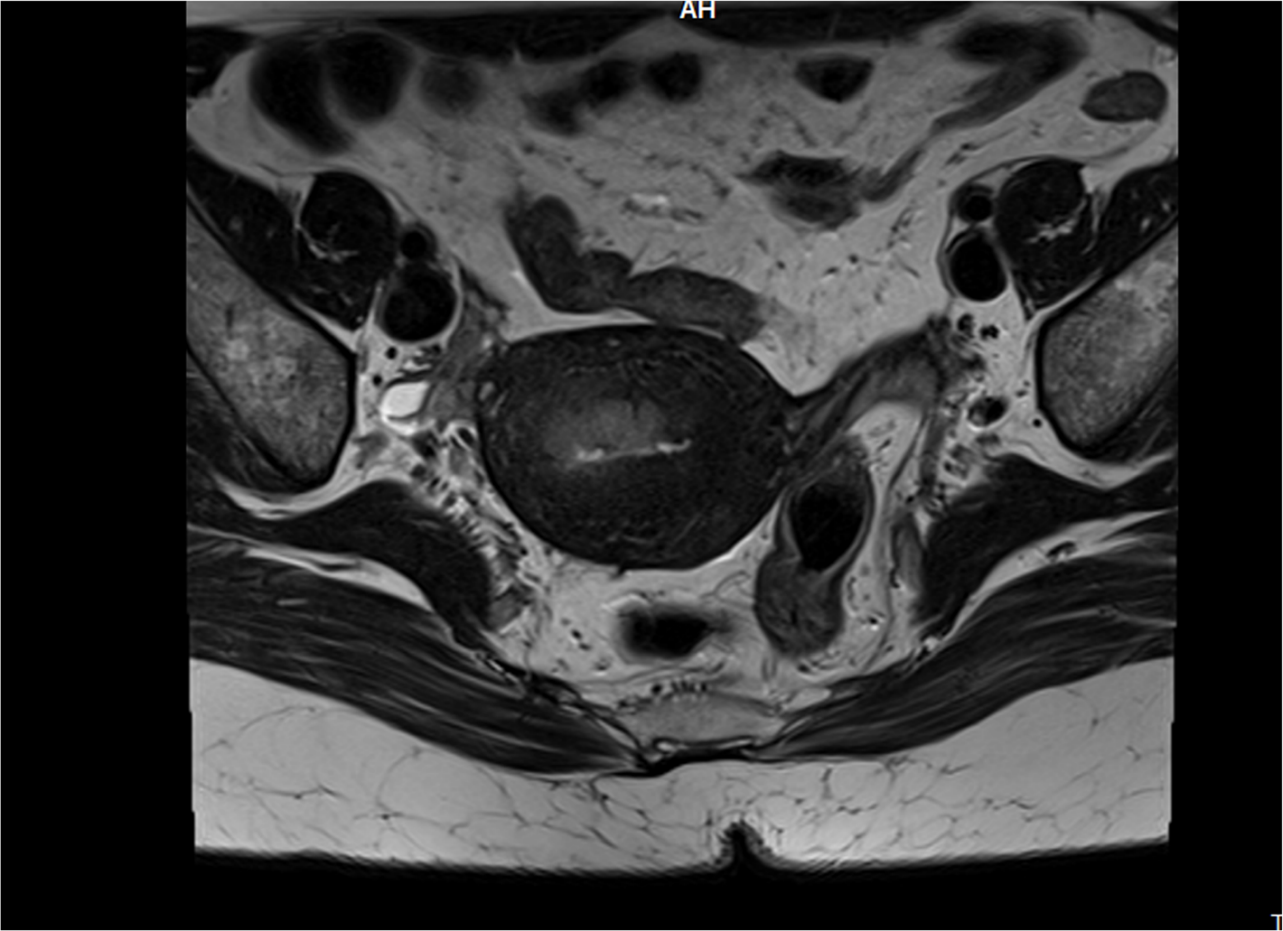
MRI showing enhancing lesion in the endometrium extending into the myometrium

**Fig. 2:**
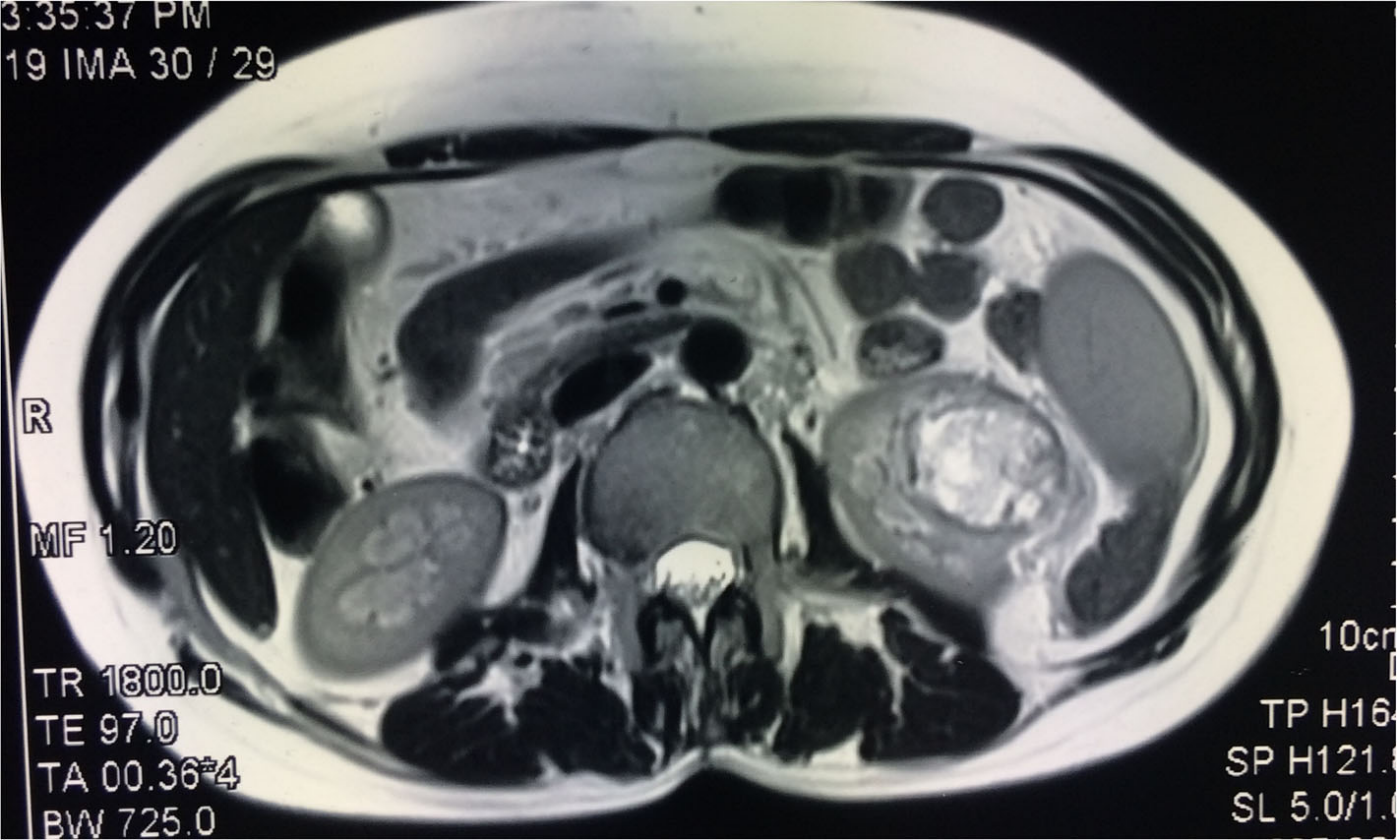
MRI (transverse section) showing enhanced renal mass at lower pole of left kidney

**Fig. 3:**
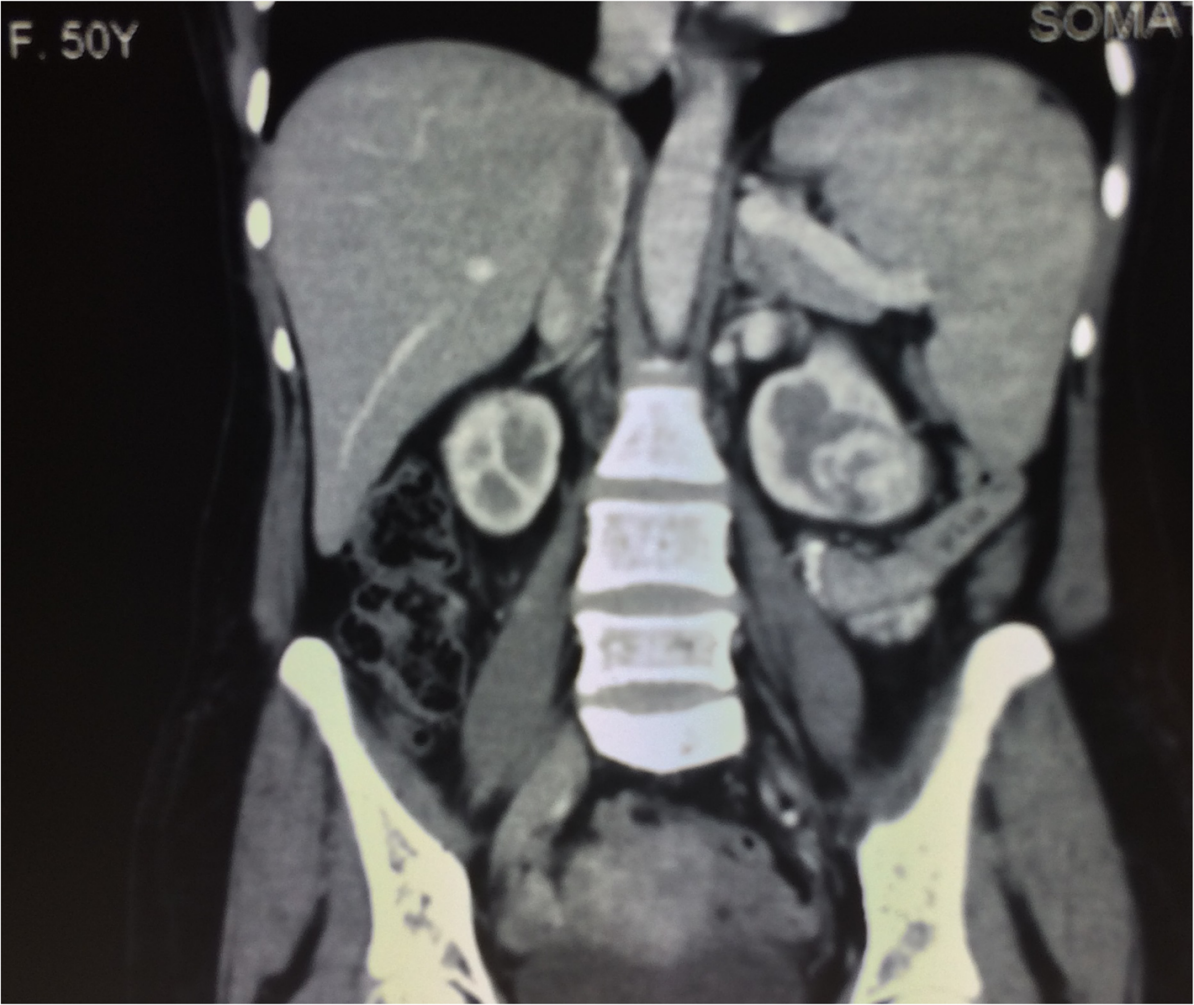
MRI (coronal section) showing renal mass reaching to pelvicalyceal system

Patient had an endometrial sampling which was reported as a high-grade endometrial adenocarcinoma (villo-nodular type). Her haemoglobin was 8.9 gm%, and kidney and liver function tests were within normal limits. S.TSH was 2.6 uIU/ml, and Ca-125 was 165 IU/L. Pap smear was reported as negative for intraepithelial lesions or malignancy. On ultrasonography (USG) of pelvis, uterus was anteverted, bulky with increased endometrial thickness with a growth within the endometrial cavity. Computed tomography revealed bulky uterus showing heterogeneous attenuation of myometrium with foci of heterogeneous enhancement in central part and a heterogeneously enhancing mass lesion towards lower pole of left kidney suggestive of mitotic aetiology most likely RCC. Contrast-enhanced MRI of abdomen and pelvis was done, and it revealed a bulky uterus with an ill-defined myometrial-based lobulated mass lesion extending into the endometrium measuring 2.4 × 1.1 × 2.0 cm with distorted endometrial cavity with adenomyosis (Fig. 1). It also picked up a 4.8 × 4 × 3.2 cm heterogeneously enhancing lesion in lower pole of left kidney reaching up to hilum and no lymphadenopathy (Figs. 2 and 3).

USG-guided FNAC from the renal lesion was done (twice); however, it yielded only blood and no tissue in both attempts. The renal mass was radiologically enhanced and vascular, so it was presumed to be a highly vascular RCC. Patient did not agree for positron emission tomography-computed tomography (PET-CT) which could have been helpful and was thus planned for staging laparotomy for endometrial cancer and frozen section for renal mass with due counselling. She underwent extra-fascial hysterectomy and bilateral salpingo-oophorectomy with pelvic lymphadenectomy and infracolic omentectomy with frozen section from renal mass. There were no ascites or omental or peritoneal deposits. As frozen section report was doubtful and inconclusive, radical nephrectomy was performed; the counselling and consent was obtained prior for the same.

**Fig. 4:**
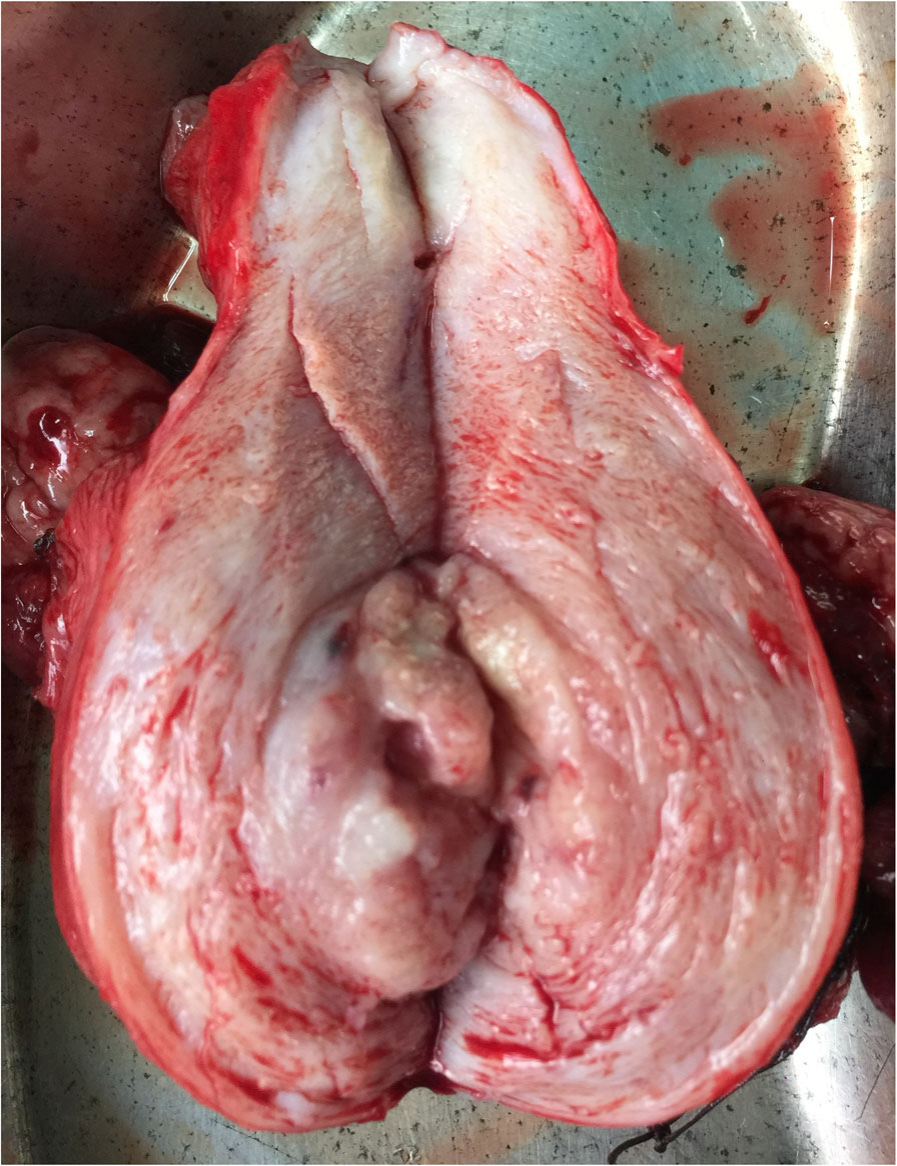
Uterus and cervix measured 9 × 5.5 × 4.0 cm; the endometrial cavity filled with papillary growth

**Fig. 5:**
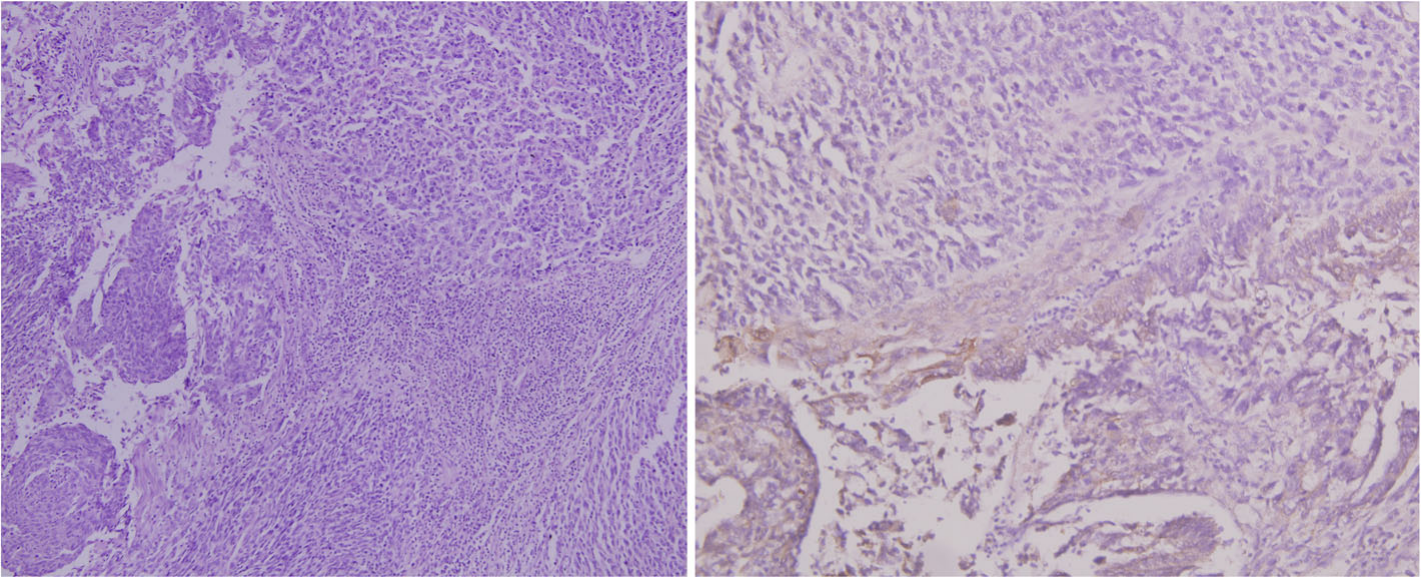
Photomicrograph of MMT showing a biphasic tumour composed of carcinomatous and sarcomatous component (H and E, × 100) and positive CK staining (IHC, × 400)

On gross examination, the endometrial cavity was filled with papillary growth 2.2 × 2 × 1.5 cm which was grossly infiltrating the outer half of myometrium with areas of necrosis (Fig. 4). There was a 4 × 5 cm well-circumscribed encapsulated growth at lower pole of kidney reaching renal hilum. Histopathological examination of the uterine growth showed a biphasic tumour with malignant epithelial component with pleomorphism, in the form of glands showing atypia and stromal component in the form of spindle cells suggestive of MMT (Fig. 5). There was no lymph vascular space occlusion, and immunohistochemistry (IHC) markers such as vimentin, keratin, desmin, myogenin, S-100, and epithelial membrane antigen were positive suggestive of MMT.

**Fig. 6:**
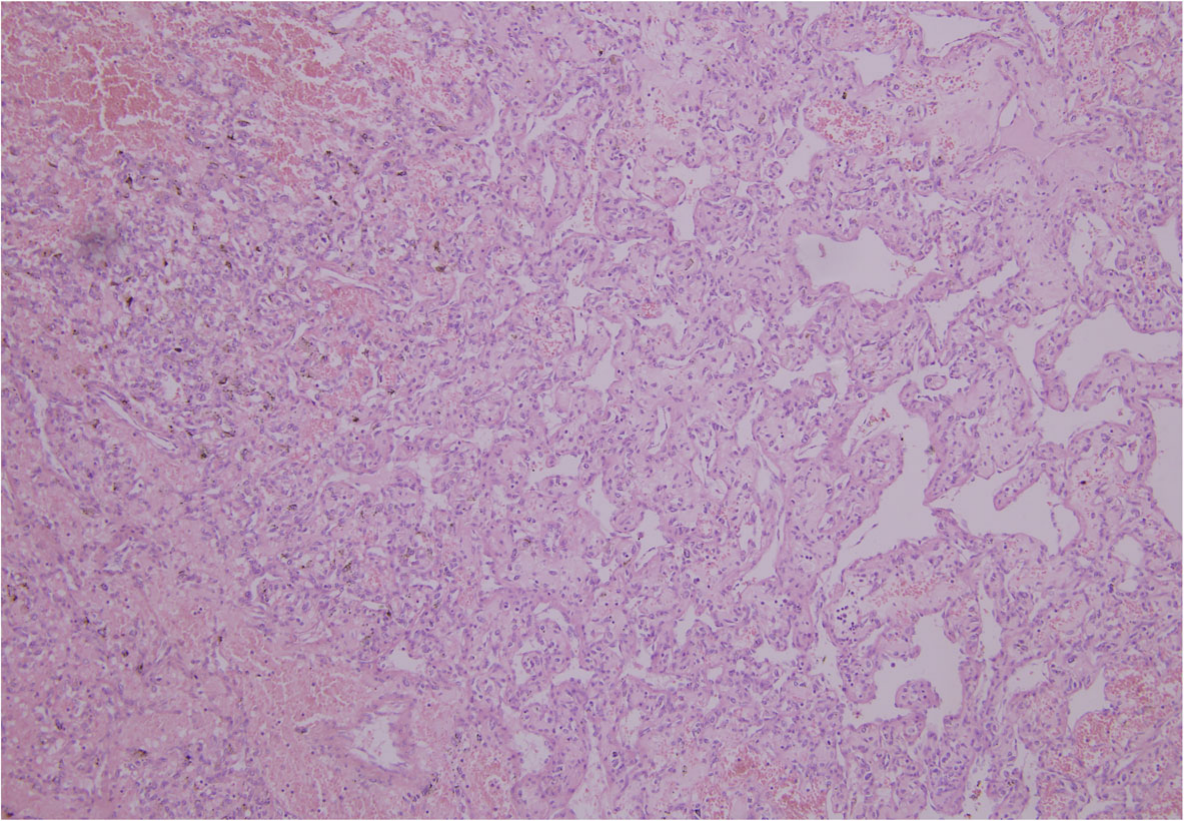
Photomicrograph of RH showing proliferation of capillaries with neoplastic stromal cells having clear to foamy cytoplasm (HE, × 100)

**Fig. 7:**
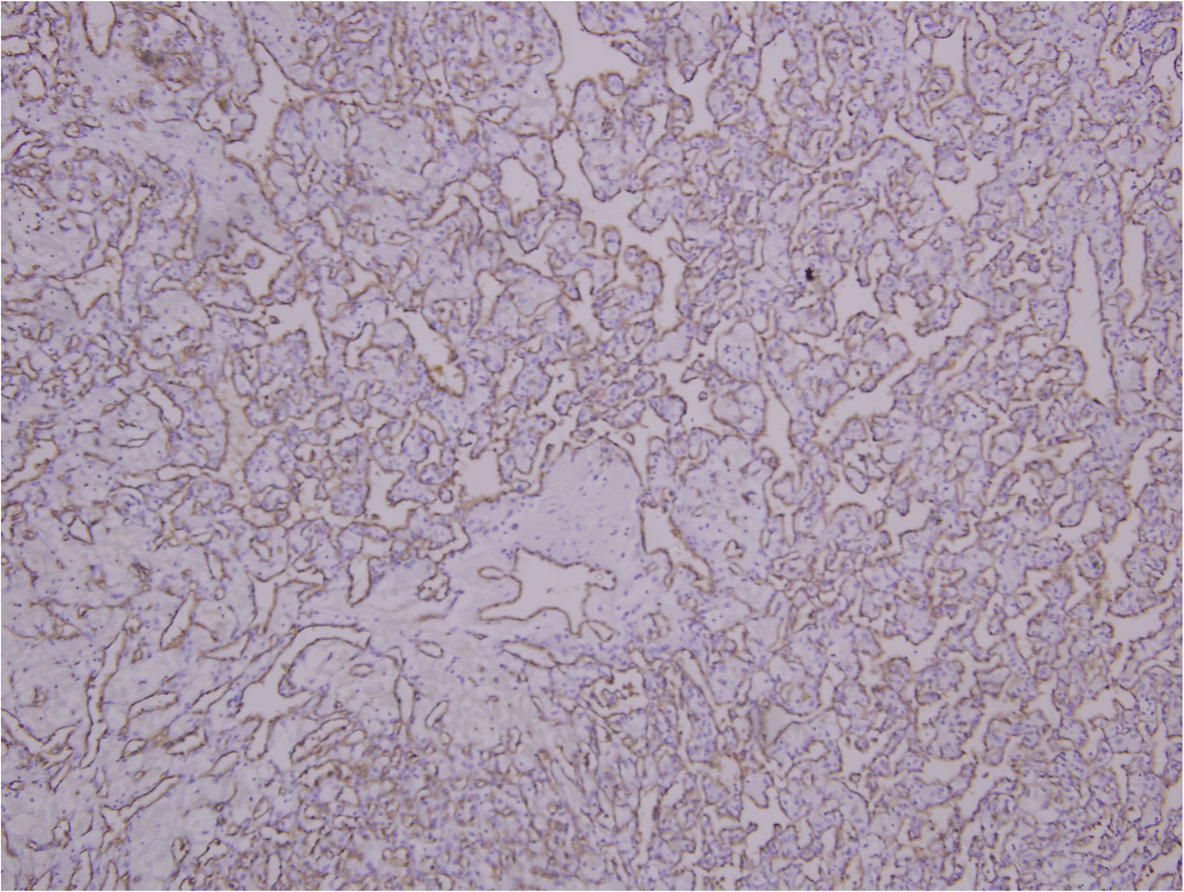
Photomicrograph of RH showing tumour cell positivity for CD34 (IHC, × 200)

**Fig. 8:**
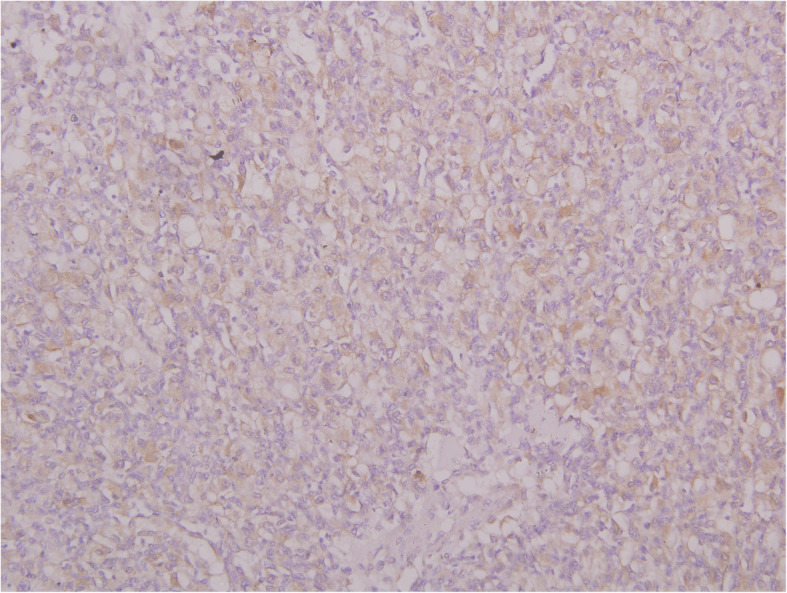
Photomicrograph of RH showing tumour cell positivity for NSE (IHC, × 400)

The renal mass showed sheets of polygonal cells with haemorrhage with minimal atypia suggestive of RH (Fig. 6). The tumour was positive for markers like inhibin, CD34, and neuron-specific enolase (NSE) by IHC and negative for vimentin and cytokeratin which are markers of RCC (Figs. 7 and 8). No vascular invasion or capsular breach was seen. Patient had uneventful recovery and was discharged on day 6. In view of RH, computed tomography head was done to rule out VHL disease, and the study came out to be normal. Medical oncologist started the patient on chemotherapy for the MMT, and she was given paclitaxel 175 mg/m^**2**^ and carboplatin AUC 5 once in 3 weeks for 6 cycles. Patient is healthy at present and is on regular follow-up for 15 months.

## Discussion

Capillary hemangioblastoma is a benign tumour which consists of a network of small blood vessels along with lipid-laden stromal cells [5]. The stromal cells may exhibit significant nuclear pleomorphism, mimicking cancer tissues. It can occur either sporadically or in association with VHL disease in around 25% of the patients [6]. The common age of presentation is between 20 and 50 years with a male to female ratio of 2:1. This tumour most commonly occurs in the CNS, predominantly in the cerebellum, but can occasionally occur in the meninges, retina, spinal cord, corpus callosum, lateral ventricle, pituitary gland, and the optic nerve. Rarely, hemangioblastoma can be seen in other sites, such as retroperitoneum, skin, liver, pancreas, lung, adrenal, peripheral nerve, soft tissue, and urinary bladder, usually in association with VHL syndrome. VHL is an autosomal dominant syndrome associated with germline mutation in the VHL tumour suppresser gene located on chromosome 3p [7, 8]. There are only few reports on sporadic hemangioblastoma occurring outside the CNS, including kidney [9–14]. To date, only 26 cases of sporadic RH have been reported. RH is an asymptomatic mass generally found incidentally as in our case. Rarely, they may present with loin pain or haematuria.

MMT also known as uterine carcinosarcoma is a rare uterine malignancy occurring in postmenopausal females in 5th to 6th decade of life. It comprises of only 1–2% of all uterine neoplasms [15]. It is a dedifferentiated and aggressive form of endometrial carcinoma having poor prognosis [16]. They have high chances of metastasis commonly to the lung (49%), peritoneum (44%), pelvic or para-aortic lymph nodes (35%), adrenal gland or bone (19%), heart or pericardium (9%), and/or brain (7%). Other rare sites include the pancreas, liver, eye thyroid gland, and skin [17]. The clinical presentation of carcinosarcomas is non-specific and is similar to other pelvic neoplasms. Patient may present with pyometra with vaginal bleeding, bloody or watery discharge, abdominal pain, or with a polypoid mass in a postmenopausal woman. A “symptom triad” has been defined favouring carcinosarcoma over endometrial adenocarcinoma which includes pain, severe vaginal bleeding, and the passage of necrotic tissue per vaginum. MMT does not have pathognomonic appearance on MRI. However, there should be a suspicion of MMT in the presence of a large heterogeneous infiltrative tumour or when tumour enhancement is equal to or greater than the myometrium. The diagnosis is made by doing endometrial sampling and HPE of the sample.

On HPE, there are both epithelial and mesenchymal elements suggestive of MMT. PET scan is useful to identify unsuspected disease and extra-pelvic site or rule out metastasis [18–20]. Surgical management should include extra-fascial hysterectomy with bilateral salphingo-oophorectomy, infracolic omentectomy, bilateral pelvic, and in some cases para-aortic lymphadenectomy.

Based on the GOG 161 study, ifosfamide/paclitaxel is considered the treatment of choice [21] and paclitaxel/carboplatin is equally effective [22] as adjuvant treatment and was used by our medical oncology team [23]. There is increasing role of adjuvant chemo-radiation now with significant overall survival; however, multiple demographic and clinical factors influence the choice of adjuvant therapy [24, 25]. Despite the use of aggressive therapy, only modest improvement in overall survival is noted over the last few decades with a 5-year overall survival rate of approximately 20–30% [26].

The occurrence of rare tumours like sporadic RH and MMT in the same patient is a very rare entity. When a new tumour is found in a patient with one existing tumour, we need to differentiate it from metastasis to determine the further management and prognosis. To differentiate, a combination of history, clinical, radiological, and pathological investigation will be needed. The most appropriate way to distinguish is by HPE as it will show different cells of origin.

RCC needs to be differentiated from RH as the former is malignant and the latter is essentially benign. Each of them has a different prognosis, and differentiating the two will help avoid over-diagnosis and unnecessary treatment.

- On radiology and gross examination, RH mimics various renal neoplasms. Clear cell variety of RCC shares various morphological features with RH, making it the most common differential diagnosis. Thus, RCC may occasionally have a hemangioblastoma-like pattern making it nearly impossible to distinguish it from RH on morphological basis [27, 28].
- The clues to the diagnosis of RH are circumscribed borders, sheets of large polygonal cells with arborizing thin-walled blood vessels and pleomorphic nuclei, and paucity of mitotic figures despite prominence of atypical cells. The presence of peri-cytomatous growth patterns and intracytoplasmic lipid vacuoles strongly suggests hemangioblastoma although both tumour types have similar morphological features, such as clear cytoplasm and a vascular network [12, 27, 28].
- To confirm the diagnosis of hemangioblastoma, IHC must be done. The absence of immunostaining for cytokeratin and positive staining for α-inhibin, S100, and NSE is diagnostic of hemangioblastoma [27, 28].
- Since they can be sporadic or associated with VHL syndrome, a polymerase chain reaction sequencing analysis of the VHL gene is done to confirm the presence of mutation in exons.

RH needs to be correctly diagnosed as sporadic RH does not require further treatment, and it has a very good prognosis [14]. Adjuvant therapy in MMT will depend on the histology of tumour in terms of local invasion, nodal status, and adequacy of margins. Prognosis in case of MMT and RH depends on biological potential of MMT as RH is a benign condition.

Such cases need a multidisciplinary approach with a team of gynaecologists, onco-surgeons, urologist, radiologist, pathologist, and medical oncologist. The key is to evaluate each tumour independently. They should be treated aggressively with the intent to cure each, so as to achieve maximum therapeutic benefit.

## Conclusion

RH is a rare benign tumour which does not require any treatment in majority of the patients. MMT is a rare aggressive uterine tumour which metastasizes early; henceforth, early identification and treatment is the key. RH needs to be differentiated from RCC to avoid over treatment. Morphological findings are similar in both; preoperative FNAC, PET scan, and intraoperative frozen section can be utilized to differentiate in between two in well-circumcised tumours and in high suspicion. The presence of two rare primary tumours, i.e. RH and MMT, in the same patient which are unrelated, is a rare entity.

## Data Availability

Not available.

## Abbreviations

MMT: Mixed Mullerian tumour
RH: Renal hemangioblastoma
MRI: Magnetic resonance imaging
USG: Ultrasonography
HPE: Histopathological examination
RCC: Renal cell carcinoma
NSE: Neuron-specific enolase
PET: Positron emission tomography
AUC: Area under curve
VHL: Von Hippel-Lindau disease
IHC: Immunohistochemistry
